# Natural variation in timing of egg hatching, response to water agitation, and bidirectional selection of early and late hatching strains of the malaria mosquito *Anopheles gambiae* sensu lato

**DOI:** 10.1186/s13071-024-06533-w

**Published:** 2024-11-20

**Authors:** Emmanuel Chinweuba Ottih, Frederic Tripet

**Affiliations:** 1 Centre of Applied Entomology and Parasitology, School of Life Sciences, Keele University, Newcastle-Under-Lyme, Staffordshire, UK; 2 Swiss Tropical and Public Health Institute, Kreuzgasse 2, 4123 Allschwil, Switzerland; 3 University of Basel, Petersplatz 1, 4001 Basel, Switzerland

**Keywords:** *Anopheles gambiae*, Egg hatching spread, Water agitation, Hatching stimuli, Hatching distribution, Diapause, Bidirectional selection

## Abstract

**Background:**

Eggs of anopheline mosquitoes hatch within a few days of laying and require high levels of humidity to survive. Assessing natural variation in egg hatching and its environmental and genetic determinants in sibling species of the malaria vector *Anopheles gambiae* s.l. is important for understanding their adaptation to variable aquatic habitats. Crucially, it can also inform insectary rearing practices toward the optimization of mosquito production for genetic vector control strategies.

**Methods:**

Hatching rates and timing of egg hatching in long-established and recently colonized strains of *An. gambiae* s.s, *Anopheles arabiensis*, and *Anopheles coluzzii*, were compared under still water conditions (26 ℃) and with cold (4 ℃) and (15 ℃) water agitation regimes. Next, early and late hatching strains of the recently colonized *An. coluzzii* VK colony were generated through bidirectional selection for 18–23 generations to detect a genetic component for this trait.

**Results:**

Hatching rates differed significantly between species and treatments. The older *An. arabiensis* Senn and *An. gambiae* s.s. Kisumu strains had the highest proportion of hatching and preferred the nonagitation treatment at 26 °C. In contrast, the more recently colonized *An. coluzzii* VK and *An. arabiensis* Rufisque strains had lower overall hatching success but responded strongly to agitation at 4 °C, while the *An. coluzzii* Mopti strain did not significantly respond to water agitation. In all strains, eggs hatching started at day 2 and continued till day 5 in the older strains, whilst it was more staggered and extended up to day 6 in the younger strains. Bidirectional selection for early and late hatching over many generations resulted in early hatching selected strains with eggs hatching 2–3 days earlier than in late hatching ones indicating a significant heritable component for these traits.

**Conclusions:**

Water agitation and temperature and age of colonization are likely important determinants of egg hatching in natural *An. gambiae* s.l. populations. Current rearing protocols systematically select for fast hatching and result in the progressive loss of staggered egg hatching in older laboratory strains. The selection of novel slow-hatching strains may prove instrumental to enable the mass production, shipping, and release of *Anopheles* mosquitoes across Africa as part of genetic vector control programs.

**Graphical Abstract:**

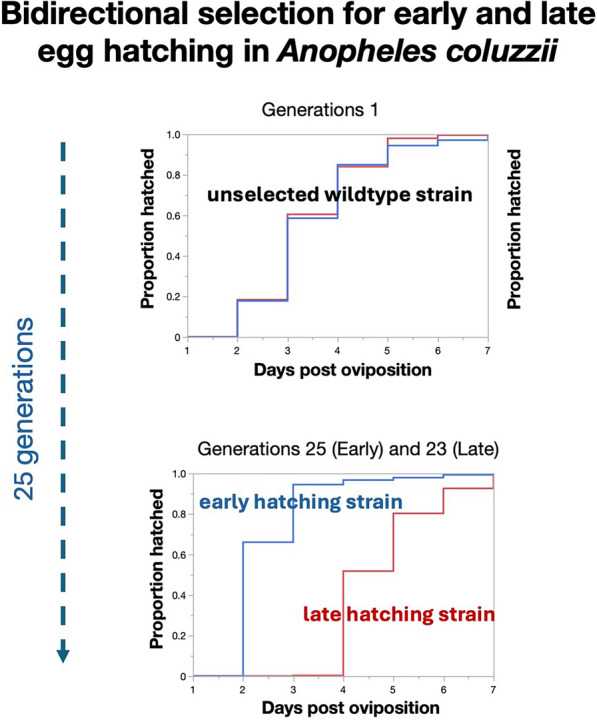

## Introduction

The *Anopheles gambiae* complex comprises some of the most important vectors of human malaria in Africa. Among the eight sibling species, *An. gambiae* sensu stricto*, Anopheles coluzzii*, and *Anopheles arabiensis* have the highest vectorial importance due to their anthropophilic nature. In West Africa, the three species are often found in sympatry in the Sudano-Sahelian zone [1, 2]. Ecological niche partitioning among this species translates into subtle differences in larval breeding site use. *An. coluzzii* density increased early at the start of the rainy season and this species is commonly associated with more permanent, man-made larval breeding sites such as rice fields [3–5]. *An. gambiae* becomes more predominant at the height of the rainy season and exploits principally the many temporary rain pools available [6]. *An. arabiensis* is typically found in drier environments where it shares temporary breeding sites, such as rain puddles and roadside ditches and with the other two sibling species [1, 6].

Currently, there is no evidence that such breeding site preference translates into differences in egg-hatching ecology such as egg-hatching time and spread but there is a notable scarcity of studies focusing on these traits [7]. In *An. gambiae* s.l., females take about 48 h to develop their eggs [8, 9]. At that stage, gravid females leave their indoor or outdoor resting sites to seek appropriate water bodies for egg laying [10]. As the eggs are laid, they are fertilized by sperm stored in the spermatheca, which travels down the spermathecal duct and penetrates the micropyle of the eggs to fertilize them [11]. After fertilization, embryogenesis ensues and it is influenced by many factors, but temperature is considered the most impactful one for early embryonic growth and survival [12]. In the early hours of embryogenesis, before the exochorion and endochorion are formed, water can still move in and out of the eggs. Within 2 h of oviposition, the exochorion darkens via melanisation, but if at that moment eggs are removed from the water, they will shrink and die [13]. In contrast, 14 h later, during mid-embryogenesis, the extraembryonic serosa cells secrete a cuticle that protects the embryo from environmental stresses by maintaining its water balance [14].

Several studies have highlighted the effect of temperature on the development of the early stages of *An. gambiae* s.s. [12, 15]. *An. gambiae* s.s. eggs maintained at high and low temperatures (42 °C and 12 °C), have reduced viability compared with those reared at 27 °C, and low temperatures slow down embryonic development [12]. Under suitable natural conditions, hatching occurs immediately after embryos have finished their development, but hatching may be delayed beyond this point when conditions are suboptimal [16]. This behavior has been recorded in different mosquito species including *An. gambiae* s.l. strains from Kenya, Congo and Mali, and Kenya [7, 17, 18]. In this species complex, delayed egg hatching is thought to be an adaptive phenotypic plastic response to the selective pressures associated with the use of temporary larval breeding habitats [7]. In arid areas, the drying of natural ephemeral breeding sites such as small rain puddles is a huge determinant of anopheline egg survival, which in turn may impact their adult population density [17]. Unlike aedine mosquitoes that can withstand desiccation over a long time, anopheline eggs need moisture to survive. *An. gambiae* s.l. eggs deposited in fast-drying environments display greatly reduced viability [17, 19]. This will also happen under humid and warm insectary conditions if eggs are not kept in water after egg laying. For example, in *An. gambiae* s.l., keeping eggs out for the water for 2 days resulted in 91% of hatching, but keeping them 12 days resulted in only 1% of eggs hatching when re-immersed [20]. These findings support the idea that in members of the *An. gambiae* complex, rain triggered, and/or staggered egg hatching, through delaying hatching of some eggs until the next rain, play key roles in an adaptive bet-hedging strategy that ensures that some reproductive success is achieved should drought temporarily affect the larval breeding sites.

Embryonic diapause is a well-known adaptation to ephemeral habitats in aedine eggs [21–23] but is rarer in anopheline species [24]. Diapause is thought to genetically programmed before it starts [25] and it is only after that fixed diapause duration has passed that eggs could respond to water stimulus and hatch [26]. The extent of egg hatching spread in the *An. gambiae* complex was explored using egg batches from wild-caught females of the sibling species of the *An. gambiae* complex collected in Mali, West Africa [7]. *An. gambiae*, *An. coluzzii*, and *An. arabiensis* all displayed very skewed distributions, with 89% of eggs hatching 2–3 days after oviposition, 10% over the next 4 days, and 1% over the next week. The exact stimuli triggering hatching in late hatching diapausing eggs are not clearly known, but two studies experimentally demonstrated that water agitation can induce egg hatching [27, 28].

In contrast to eggs from natural populations, laboratory strains of *An. gambiae* s.l. tend to hatch more synchronously as standard rearing practices may systematically select against late hatching eggs. This is because first instar larvae are typically placed in trays 1 or 2 days after emergence and therefore originate from the more numerous fast hatching eggs while slower hatching eggs and progeny are often discarded. Thus, one would predict that over time, laboratory strains are selected for early egg hatching and reduced hatching spread. Whether there was a heritable component controlling the time of hatching of *An. gambiae* eggs was investigated through the selection of early and late hatching lines which resulted in an 30% increase in the proportion of late hatching larvae after six generations [27]. The result of early × late hatching strain crossing experiments suggested that egg-hatching time did not follow a simple Mendelian mode of inheritance [27]. Furthermore, the rate of metabolic activity and embryonic development did not differ between early and late hatching lines [18].

Understanding the environmental and genetic basis of egg hatching is particularly relevant to novel approaches to malaria control via mosquito release programs. Over the last few years, the reduction in malaria transmission achieved through classic vectors chemical control intervention chemicals such as insecticide-treated nets (ITNs) and indoor residual spray (IRS) have significantly slowed down [29]. Continued reliance on chemical tools is proving ineffective due to the spread of multiple resistance to all classes of insecticide used for public health in mosquito populations, as well as changes in their behavior that decrease the impact of those interventions [29]. The limitation of chemical vector control approaches is driving an important research effort focusing on improving the sterile insect technique (SIT) for anopheline malaria vectors, with a particular focus on novel genetic approaches relying on heritable gene constructs passed on from one generation to the other [29, 30]. Heritable approaches involve the releasing of large numbers of genetically modified mosquitoes into the target mosquito population. They are considered self-limiting when the genetic constructs are inherited in a Mendelian fashion and disappear from the target population within a few releases, or self-sustaining for those that persist in the target population for a longer period, thanks to biased gene construct inheritance [29, 30]. For SIT and self-limiting genetic approaches, there is a need to release large numbers of males for an extended period to achieve target population suppression [30]. Given the size and complexity of the targeted *An. gambiae* s.l. populations, a self-sustaining approach would require considerable mosquito production capacity [30, 31].

Mass mosquito rearing will necessitate facilities and processes of mosquito production, transportation, and release that preserve mosquito fitness [32]. When target populations are dispersed and remote, egg-to-adult rearing facilities that receive bulk egg shipments from larger production centers may be the best option for mosquito distribution. However, the shipment of standardized numbers of anopheline eggs is currently difficult due to their intolerance for a prolonged period of drying, and the tendency to stick together when fresh [33]. The impacts of drying and the use of different storage methods on *An. gambiae s.s* and *An. arabiensis* egg hatchability, larval development, and survivorship have been explored through various studies [12, 33–35]. Through elaborate drying and storage methods, combined with narrowly controlled humidity and temperature conditions, storage times of up to 4–6 days have been achieved without negative impacts on hatch rate and larval development. However, beyond that point, all available studies found that hatch rate and larval survivorship decreased with storage time, regardless of the storage method employed.

Considering the important bottleneck that natural egg-hatching behavior in malaria vectors constitutes for release programs, there is a need for a better understanding of its ecology and genetics. In this study, the impact of water agitation at 4 °C and 15 °C and still water at 26 °C on hatch rate and distribution hatching time were investigated in three long-established *An. gambiae s.s*, *An. arabiensis*, and *An. coluzzii* strains and two more recently colonized strains of *An. arabiensis* and *An. coluzzii*. Next, the strain with the highest level of egg hatching spread was used in replicated bidirectional selection experiments for ~20 generations, resulting in two early and late hatching strains. These results are important for our understanding of egg-hatching genetics and the behavior of *An. gambiae* complex with implications for Anopheline production toward release strategies for malaria control.

## Methods

### Strains and maintenance

The five strains belonging to three sibling species of *An. gambiae* complex used in this study were: *An. arabiensis* Senn colony established in 1969 from Sudan and the Rufisque strain established in 2017 from Rufisque Senegal; *An. gambiae s.s* Kisumu strain, established from Kisumu, Kenya in 1975 [36], *An. coluzzii* Mopti strain colonised in 2003 from N’Gabakoro Droit, Mali in West Africa [37], and the more recently colonized *An. coluzzii* VK strain established from Vallee du Kou, Burkina Faso in 2017. Two months before the start of the study, special attention was paid to not discarding late hatching eggs of the younger strains such as the VK and Rufisque, so as not to select for early hatching ahead of the experiment. late hatching larvae were therefore also trayed out and reared to adulthood to contribute to the next generations. The five strains were maintained at a temperature of 26.5 ± 1 °C and 75 ± 5% relative humidity (RH), under a photoperiod of 12:12 (light: dark) hours cycle. The larvae from all strains were reared at a density of 200 per tray (34 × 24 cm) in 1 L of water and maintained on ground fish food (Tetramin, Tetra, Melle, Germany) from first to third instars, and Koi food (Tetra Pond sticks, Tetra GmbH Germany) and carnivore fish pellets (Kyorin Food Ind Ltd) thereafter. On pupation, the pupae were transferred into a 100 ml polystyrene cup containing tap water and placed in the center of a cage for adults to emerge. The emerged adults remained in the mosquito-netted roof cages made of 5 L white polypropylene buckets (19.5 cm height by 19 cm diameter), and an opening by the side with a sleeve for access to the interior of the cages. Adults were always provided with a 10% glucose solution.

### Collection and preparation of eggs

For egg collection, about 100–150 females were fed on defibrinated horse blood using an Hemotek membrane feeding system (Discovery Workshops, Blackburn, UK). Three days after blood feeding, gravid females were given an oviposition cup. The cups containing water were placed in cages from 15:00 to 16:00 and removed gently from the cages between 9:00 and 12:00 the next day (referred to as day 1 after oviposition). Eggs were transferred from the oviposition cups into experimental Petri dishes (13 mm height by 60 mm diameter) holding 6–10 ml of mixed (mineral and deionized) water using a toothpick. The toothpick was inserted into the cup holding the eggs and moved towards the eggs until they adhered to it. Twenty eggs were placed into each Petri dish, and they were closed with a lid to avoid evaporation. A total of 7200 eggs were used in the study.

### Variation in hatching time experiments

#### Experimental design

The effects of water agitation (nonagitated “still” water or daily “spray”) and, for the sprayed groups, the temperature of sprayed water (cold 4 °C or 15 °C) on hatch rates of *An. gambiae*, *An. arabiensis*, and *An. coluzzii* (Mopti and VK strains) were tested over 7 days. Each of these three treatments was replicated three times using different mosquito generations: three different batches from each of the species (strains). Fifteen Petri dishes (30 ml each) were prepared for each replicate, with each containing 20 eggs of the species to be tested resulting in 600 eggs used per replicate. Petri dishes were regularly assigned to each of the three treatments and spatially alternated on trays on the insectary bench surfaces to avoid positional bias due to subtle variation in the insectary environment.

### Daily agitation with 4 °C and 15 °C water and hatch rates

We used the method described by Ebrahimi et al. [28], with slight modifications to agitate the water by spraying and measuring hatch rates. Ten minutes after distributing eggs into Petri dishes on day 1, the first water agitation was conducted by spraying 4 °C and 15 °C water into the Petri dishes; this was then repeated daily for another 6 days. The spraying of water was done using a wash bottle in an anticlockwise direction. Petri dishes from the still group did not receive any spray. Every day, larvae were counted by eye and removed with a Pasteur pipette before spraying and 1 h after spraying, taking all precautions not to generate unnecessary disturbances that could cause hatching. Before spraying, each of the Petri dishes to be treated was gently uncovered, placed on the bench, and then sprayed gently for 5–10 s to avoid spilling out of eggs and overflooding. Excess water was constantly removed every day before treatment. Any eggs that stuck to the walls of the dishes were washed back onto the water with one or two drops of water using a Pasteur pipette. For the unsprayed still group, the mixed (mineral and deionized) water was replenished when the level was low to compensate for the loss of water due to the removal of hatchlings. The final inspection was conducted on day 7, 1 h after the final spray treatment.

### Bidirectional selection experiment on VK strain

#### Experimental design

Prior to the selection experiment, the VK strain was refreshed genetically by crossing virgin males and females with the progeny of field-collected *An. coluzzii* females from the same location. The bidirectional selection experiment was initiated using two egg batches obtained from the second generation of the refreshed VK strain. The study was replicated resulting in two independently selected pairs of early and late hatching strains (see selection of phenotypes). The two replicates are referred to as replicate 1 and 2 throughout the text.

### Preparation of eggs

Methods for egg collections and preparations were similar to those used in the first experiment, except for a higher number of eggs used and the use of a single set of conditions for hatching.

Sixty Petri dishes (13 mm height by 60 mm diameter) holding 6–10 ml of mixed water were prepared for each generation with each containing 30 eggs for a total of 1800 eggs in each batch, giving a total of 54,000 eggs. Each of the trays containing the dishes had ten Petri dishes and were placed on the same bench to assume equal effects. The Petri dishes containing eggs were agitated by gentle spraying of cold water (4 °C) daily for 7 days starting 1 h after preparation. Cold water was used because the VK strain responded better to cold water compared with normal water in the “variation in hatching time” experiment. Water was sprayed in an anticlockwise direction for 3–10 s and the hatchlings were collected 1 h after the treatments.

#### Selection of phenotypes

The early and late hatching lines for the two replicates were established from their parent generations (generation after the refreshment). Eggs obtained from the parent generation were prepared as described above. The larvae that hatched on the second day were counted, collected, and considered the early hatchers, while the ones that hatched on the third day were counted and culled. Additionally, larvae that hatched from day 4 to 7 were collected, counted, and labeled late hatchers. Larvae from both early and late hatching phenotypes were reared separately under the same conditions, with each batch (parents) providing two lines (early and late hatchers) making up a total of four lines from the two different batches with each line containing approximately 400 mosquitoes.

After the first pair of early and late lines had been established from the parental generation, the same procedure was repeated from the first generation of the selected phenotypes. From each selected line, the larvae producing early and late hatching selected lines were used to establish the next generation. Larvae that hatched later than the second day in the fast-hatching selected line were counted and culled out. The same applied to the slow-hatching ones—any fast (early) emerged larvae were counted and culled out to maintain strictly late hatchers. The bidirectional selection was imposed for 23 (late strain) and 25 (early strain) generations for the first replicate, and 17 (late strain) and 18 (early strain) generations for the second.

### Statistical analysis

All analyses were carried out using JMP 14.0 software (SAS Institute, Inc., Cary, North Carolina, USA). The datasets were inspected for normality in distribution and heterogeneity of variance. The relative effects of mosquito strains and water treatments on the egg hatching rate (binomial variable) were compared using nominal logistic regression, followed by post hoc pairwise comparisons between groups using likelihood odds ratios. Egg hatching timing was compared between strains and treatments using Cox proportional hazard models, followed by pairwise comparisons based on hazard ratios. Replicate effects were tested in all models but only included when significant. Kaplan–Meier curves and Wilcoxon tests to describe differences in the distribution of egg hatching times in both parents and the selected phenotypes.

## Results

### Variation in hatch rates between strains

A total number of 9000 eggs were used over three replicated comparisons of the five strains (1800 eggs per strain) (Table 1). A logistic regression model showed that the strains differed significantly in their hatching rates (Table 2). Pairwise post hoc comparisons revealed that the hatch rates of *An. arabiensis* Senn and *An.* gambiae Kisumu were significantly higher than other strains (likelihood odds ratios: *P* < 0.001 in all cases) but that they did not differ significantly from one another (likelihood odds ratio: odds ratio = 1, *P* = 0.847). *An. coluzzii* Mopti had an intermediate hatching rate, while the hatch rates of the recently colonized *An. arabiensis* Rufisque and *An. coluzzii* VK were significantly lower compared with all others (odds ratios > 3.2; *P* < 0.001 in all cases) (Table 1, 2).

**Table 1:** Mean egg hatch rate (standard deviation) in relation to water treatment in the five strains from three sibling species of *An. gambiae* complex, including *An. arabiensis* strains Senn and Rufisque, *An. coluzzii* Mopti and recently colonized VK strains, and *An. gambiae s.s.* Kisumu

Treatments	Still	4 °C Spray	15 °C Spray	All
Strains	% Hatched (SD)	% Hatched (SD)	% Hatched (SD)	Mean % hatched
Senn	88.2 (5.1)	78.8 (4.3)	82.7 (3.4)	83.2
Rufisque	34.2 (5.9)	67.5 (14.8)	63.7 (9.3)	55.1
Mopti	77.00 (10.0)	76.5 (6.1)	77.3 (0.8)	76.9
VK	20.2 (11.4)	68.5 (10.1)	66.0 (6.1)	51.6
Kisumu	90.2 (9.5)	70.8 (10.7)	84.0 (2.6)	81.7
All strains	61.9	72.4	74.7	69.7

**Table 2:** Logistic regression (likelihood-ratio tests) testing the effects of strains, replicates, and treatments and their respective interactions on egg hatch rates

Source	Degrees of freedom	Chi-squared	*P*-value
Strains	4	755.97	< 0.001
Replicate	2	19.39	< 0.001
Treatments	2	40.94	0.001
Strains × replicate	8	136.41	< 0.001
Strains × treatments	8	513.98	< 0.001
Replicate × treatments	4	30.96	< 0.001

### Effects of daily agitation with 4 °C and 15 °C water on hatch rate

There was a strong overall effect of water treatments on hatch rates with still water at 26 °C inducing the lowest hatch rates and water agitation at 4 °C and 15 °C the highest rates (Table 1). Pairwise comparisons of treatments showed that they all differed significantly from each other (odds ratios > 0.6, *P* < 0.011 in all cases). There was a significant main effect of replicate on hatch rates and strong significant interactions between strains and water treatments (Table 2).

**Fig. 1:**
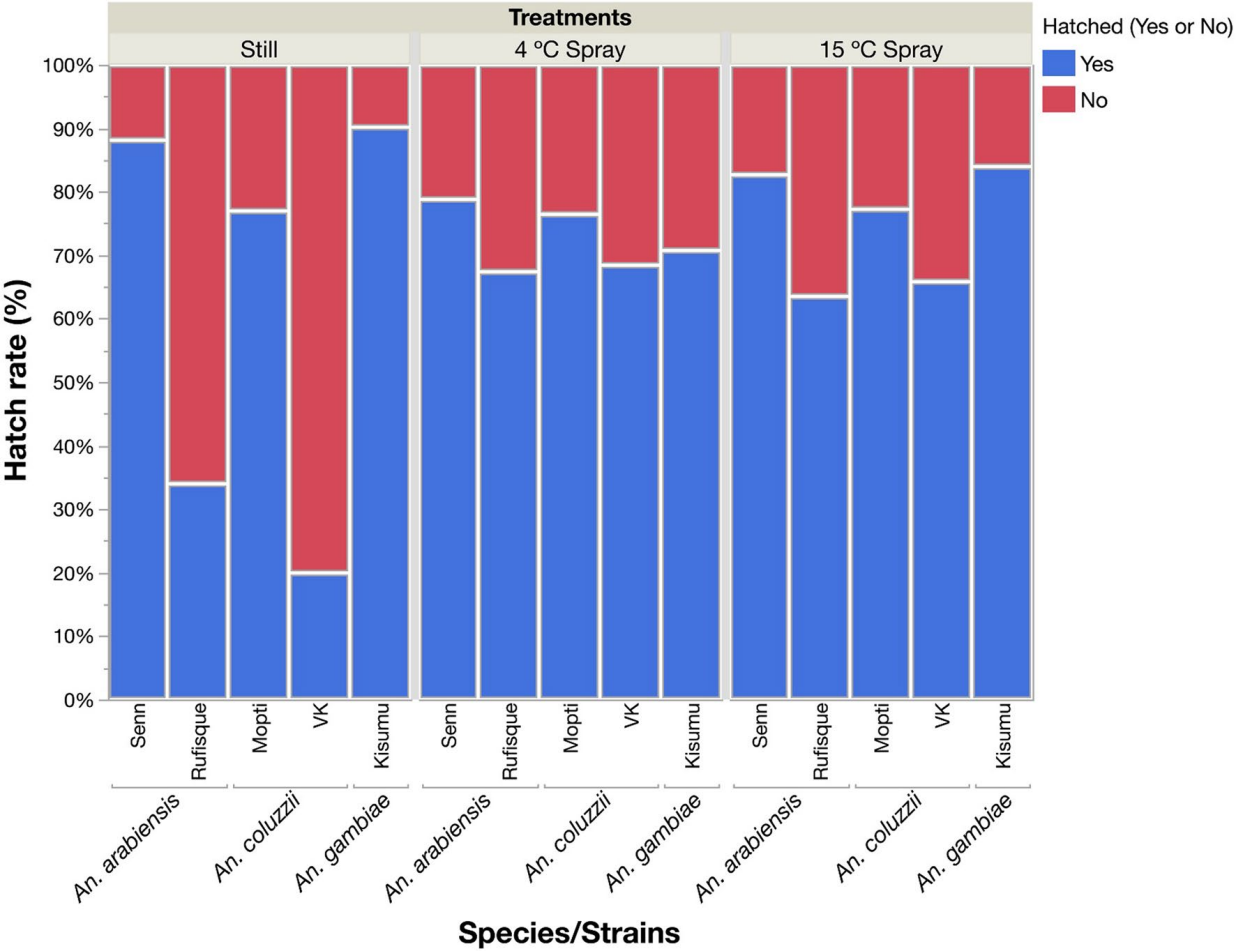
Percentage of eggs that hatched in relation to water agitation and temperature in older (Senn, Mopti and Kisumu) and recently colonized (Rufisque and VK) strains of the *An. gambiae* complex

We further explored the interaction between water treatment and strain by rerunning independent logistic regression models for each strain or each water treatment followed by post hoc pairwise comparisons. In the Mopti strain, there was no significant effect of water treatments on hatch rates (odds ratios > 0.9; *P* > 0.731 in all cases) (Table 2; Fig. 1). For the older Senn and Kisumu strains, and the younger Rufisque and VK strains, the treatments had significant but opposite effects on the hatch rates (Table 2, Fig. 1). Senn and Kisumu recorded 7.5% and 12.8% increases in hatch rates in still water (odds ratios > 1.5; *P* < 0.001 in both cases); in contrast to Rufisque and VK whose hatch rates decreased by 31.4% and 46.8% in that group (odds ratios > 3.3; *P* < 0.001 in both cases) (Table 2, Fig. 1). The Mopti and VK strains are the same species (*An. coluzzii*) but were colonized at different times. However, in still water, eggs from the recently colonized strain VK were less likely to hatch without agitation compared with those of the older Mopti (odds ratio = 13.3; *P* < 0.001) (Table 2, Fig. 1). A similar pattern was observed between Rufisque and Senn, the eggs of the former being less likely to hatch without water agitation (odds ratio = 14.4; *P* < 0.001). The VK and Rufisque strains were also less likely to hatch in still water compared with the *An. gambiae* Kisumu (odds ratios > 17.6; *P* < 0.001 in both cases) (Table 2, Fig. 1).

### Effects of strain and water treatment on egg hatching time

**Fig. 2:**
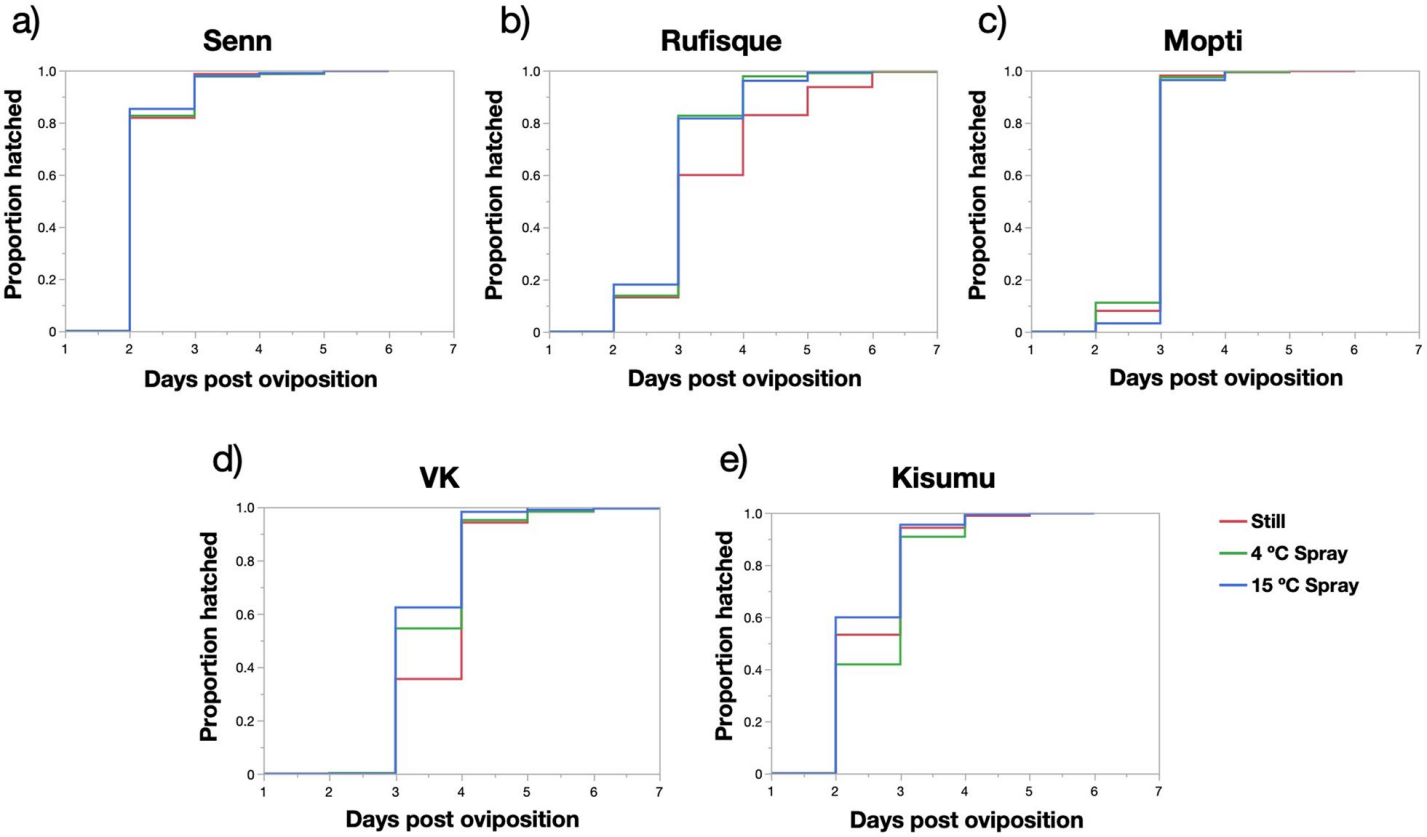
**a–e** Kaplan–Meier plots of egg hatching time spread in relation to experimental water treatments in five strains of *An. gambiae* differing in age of colonization: **a**, **b** *An. arabiensis* Senn and Rufisque, **c**, **d** *An. coluzzii* Mopti and VK, (**e**) *An. gambiae s.s.* Kisumu

Across all water treatments, no eggs hatched within 24 h after oviposition. In the oldest strains, Senn, Kisumu, and in the Mopti strain, most of the eggs hatched within the first and second days (Fig. 2). Egg hatching was staggered over as much as 5 days in all three species, but the latest hatching time recorded for the older strains was on day 5, while in the most recently colonized and selected strains, VK and Rufisque, respectively, eggs staggered up to day 6 (Fig. 2). The proportional hazard model revealed that the timing of egg hatching significantly differed between strains, water treatments, and replicates (Table 3). There was also a significant interaction between the effect of strains and water treatments (Table 3). The more recently colonized VK and recently selected Rufisque strain were significantly less likely to hatch early on (day 1 or 2 postoviposition) and hatched over more days compared with the older Senn, Mopti and Kisumu strains [hazard ratios (HRs) > 1.5; *P* < 0.001 in all cases) (Table 3, Fig. 2). Senn was the oldest strain in this study and 69% of its eggs hatched on day 1 compared with 0.06% for VK and 8.4% Rufisque (HRs > 2.9; *P* < 0.001 in both cases) (Table 3). Comparing the *An. coluzzii* Mopti and VK strains revealed that the older Mopti was nearly 100-fold more likely to hatch on day 1 than the VK (HR = 1.5;* P* < 0.001).

**Table 3:** Proportional hazards analysis testing the effects of strains, replicates, and treatments with their respective interactions on the egg-hatching time of the five strains tested

Source	Degrees of freedom	Chi-squared	*P*-value
Strains	4	1050.31	< 0.001
Replicate	2	5.48	< 0.001
Treatments	2	11.77	0.031
Strains × replicate	8	96.8	< 0.001
Strains × treatments	8	25.59	0.019
Replicate × treatment	4	19.24	< 0.001

There was no overall significant difference between the still and 4 °C agitation treatments (hazard ratios = 1.0; *P* = 0.214) but water agitation at 15 °C significantly accelerated hatching compared to 4 °C and still water (HRs > 0.9; *P* < 0.019 in both cases). As the effect of water treatment drastically differed for each strain, independent proportional hazard models were run for each strain independently to highlight their specific responses to water agitation at 4 °C and 15 °C. Water treatments did not significantly affect the Senn and Mopti strains (HRs: Chi-squared < 1.6, *P* > 0.446 in both cases) but moderately affected the Kisumu strain (Chi-squared = 8.4, *P* = 0.015) and VK (Chi square = 6.4, *P* = 0.039) and strongly affected Rufisque (Chi-squared = 17.1, *P* < 0.001) (Fig. 2).

### Bidirectional selection experiment on VK strain

**Fig. 3:**
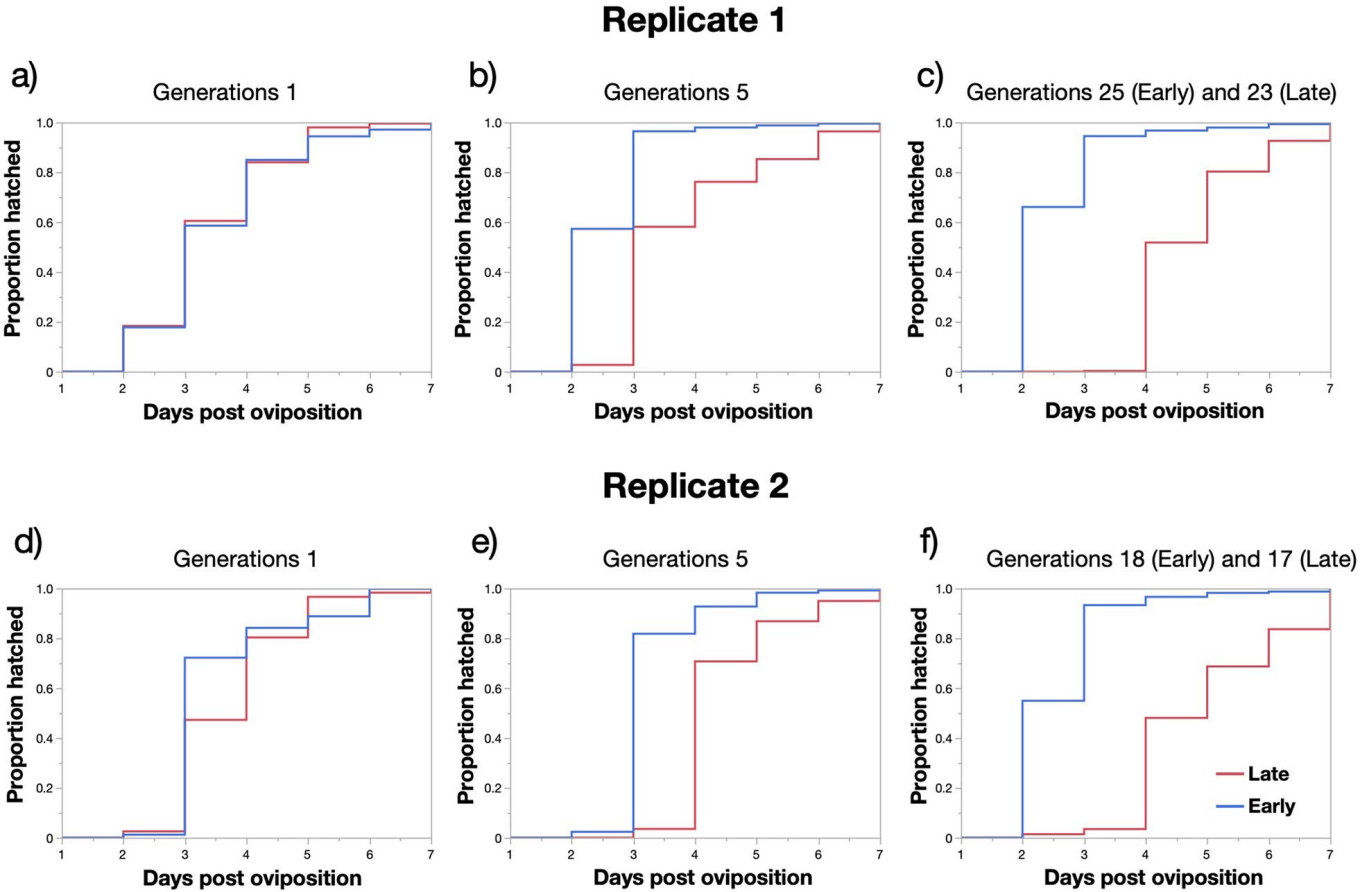
**a–f** Kaplan–Meier plots of egg hatching time spread in unselected, fifth generation-selected, and later generations of bidirectionally selected early (red) and late (blue) hatching phenotypes **a**–**c** replicate 1; **d**–**f** replicate 2

The bidirectional selection extended up to 23rd or 25th generations in the first replicate and 17th or 18th generations in the second replicate. From the F_2_, the selected early hatching strains hatched significantly earlier than late hatching ones. Moreover, as generation progressed the difference in mean and median time of hatching between the two phenotypes increased (Fig. 3, Table 4). At the end of study, the minimum median hatching times for the early and late hatching groups were day 2 and 4 in the first replicate respectively and day 2 and 5 in the second replicate (Fig. 3, Table 4). By generations 25 (repl. 1) and 18 (repl. 2), the eggs of early hatching strains also hatched significantly faster than those of the starting F_1_ generation (Kaplan–Meier Wilcoxon: Chi-squared > 660.4, *P* < 0.001 in both cases). Similarly, the timing of egg hatching of both late strains at generation 23 (repl. 1) and 17 (repl. 2) differed significantly from late hatching eggs from the F_1_ generation (Chi-squared > 438.0, *P* < 0.001 in both cases).

**Table 4:** Means and median egg hatching times (days) and results of Kaplan–Meier Wilcoxon tests comparing the early and late hatching phenotypes at different generations for both replicates

Generation	Replicate	Group	*N*	Mean time	Median time	Wilcoxon Chi-squared	*P*-value
1	1	Early	1035	3.47	3	0.539	0.463
		Late	850	3.39	3		
	2	Early	993	3.54	3	72.56	< 0.001
		Late	1062	3.75	4		
2	1	Early	943	3.22	3	81.42	< 0.001
		Late	571	3.62	3		
	2	Early	991	3.56	3	51.21	< 0.001
		Late	1151	3.93	4		
3	1	Early	1084	2.99	3	304.10	< 0.001
		Late	873	3.76	3		
	2	Early	1132	3.44	3	9.236	< 0.001
		Late	1210	3.38	3		
4	1	Early	966	3.03	3	244.80	< 0.001
		Late	1206	3.44	3		
	2	Early	1183	3.44	3	204.08	< 0.001
		Late	892	4.09	4		
5	1	Early	1419	2.50	2	958.27	< 0.001
		Late	964	3.81	3		
	2	Early	1226	3.26	3	1193.03	< 0.001
		Late	994	4.44	4		
6	1	Early	987	3.43	3	698.38	< 0.001
		Late	1071	4.56	4		
	2	Early	1278	2.85	3	829.58	< 0.001
		Late	1082	3.96	4		
24 and 25	1	Early	1302	2.45	2	1898.87	< 0.001
		Late	1071	4.75	4		
17 and 18	2	Early	1118	2.58	2	1201.09	< 0.001
		Late	722	4.94	5		

**Fig. 4:**
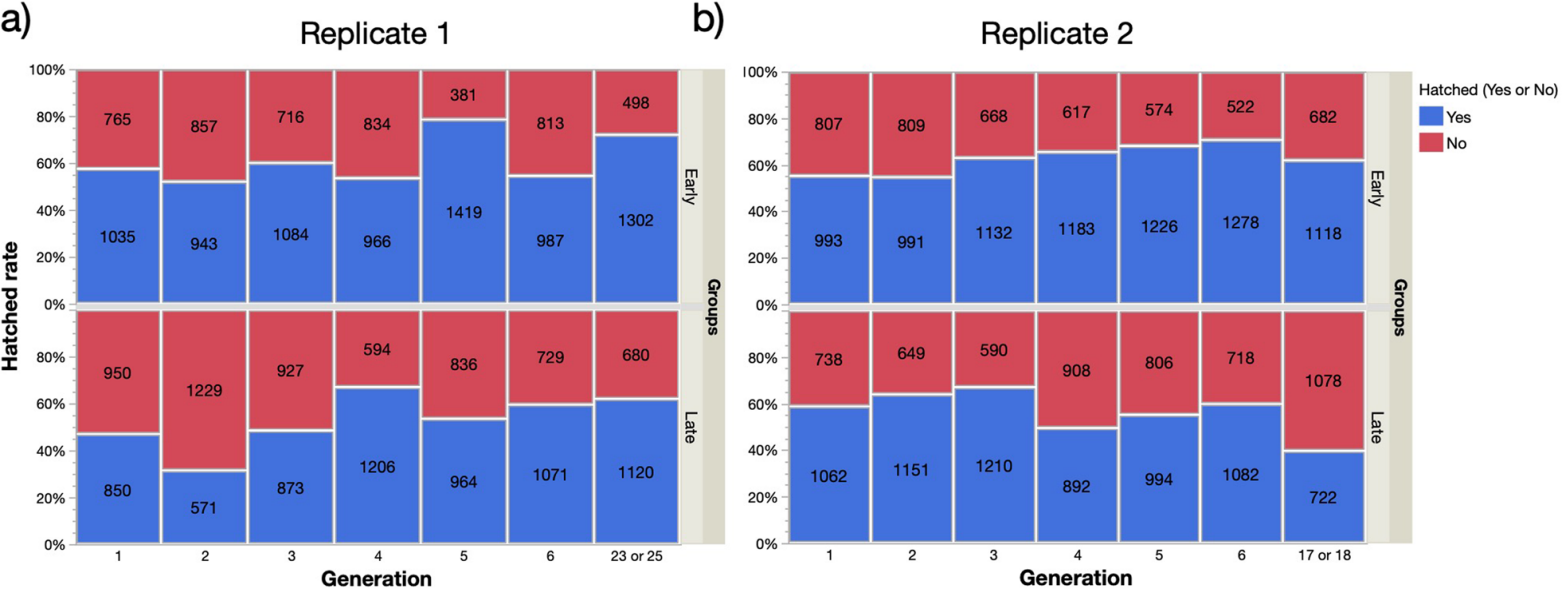
**a,b** Egg hatching rates of late and early hatching strains for different generations: **a** hatch rate of replicate 1 for different generations; **b** hatch rate of replicate 2 for different generations

Over all generations, late strains had a significantly lower hatching rate than Early ones (Likelihood ratio chi-squared:  > 107.7, *P* < 0.001 in both replicates). For the first replicate, hatching success was 61.4% and 52.8% for the early and late hatching strains, respectively; for the second replicate, 62.9% and 56.5% for early and late strains, respectively (Fig. 4). Logistic regression revealed that hatch rates also significantly improved as the number of generations of selection increased and that they were also significantly higher for the strains generated in replicate 2 (Table 5). Significant interactions between the effect of replicate, selection group and generation highlighted considerable variation in data (Fig. 4, Table 5).

**Table 5:** Logistic regression (likelihood-ratio tests) testing hatch rate in relation to selected groups (early or late hatching phenotypes), generations, and replicates and their respective interactions

Source	Degrees of freedom	Chi-squared	*P*-value
Groups	1	7.56	0.006
Generation	1	660.9	< 0.001
Groups × generations	1	173.5	< 0.001
Replicates	1	16.2	< 0.001
Generation × replicates	1	253.67	< 0.001

## Discussion

The results from this study demonstrate that anopheline eggs from old and newly colonized strains of the *An. gambiae* complex exhibit significant differences in their hatching patterns. The longer the time since colonization, the more likely their eggs were to hatch early and without the need for water agitation. Long-established strains also had higher overall hatch rates and hatching staggered over fewer days. These results are compatible with the expectation that standard laboratory rearing practices select for eggs that hatch early and without the need for the stimulus of, e.g., rainfall.

The larger egg hatching spread and variability in response to water agitation at 4 °C and 15 °C in the recently colonized VK and Rufisque strains is consistent with field observations that report that eggs laid on open water bodies can either hatch spontaneously after embryogenesis or delay hatching until induced by mechanical stimulation [12, 38]. In this study, we observed that most eggs hatched within 1 h of agitation, more hatched as first instars larvae were being picked from Petri dishes, and a few still hatched later. There is clearly variation in the amounts of water agitation required for eggs to hatch or variation in the embryonic stage of development, which causes a delay in hatching in some eggs, but not in others. These results suggest that under natural conditions the water agitation resulting from raindrops is an important cue triggering some eggs to hatch as an adaptive response to improving conditions for the larval development. Given that *An. gambiae* s.l. often uses small ephemeral breeding habitats such as small rain puddles and ditches [39], such a response could improve the larvae’s chances of completing their development or reaching a nearby larval site before the habitat dries out [15]. Having a mixture of eggs hatching early and others hatching in a rain-dependent manner may be part of an adaptive bet-hedging laying strategy by *An. gambiae* s.l. females, which are known to use various aquatic habitats that vary drastically in terms of availability and in the proportion of ephemeral to more permanent ones depending on the season and local environment [39].

In our older colonized strains, this rain-dependent adaptive response to agitation seemed to have been lost due to the selection pressures associated with insectary rearing [37, 40]. The Kisumu, Senn, and Mopti strains had their highest hatch rates without physical agitation at 90.2%, 88.2%, and 77.0%, respectively, which contrasted with the recently colonized VK and Rufisque strains whose hatch rates without water agitation, at 20.2% and 34.2%, were very low. Due to insectary maintenance, colonized strains may be commonly selected for early hatching due to simplified rearing protocol wherein early hatchers are inadvertently and constantly selected for because late hatching eggs are often discarded, or the larvae hatching from them do not develop in time to significantly contribute to the next adult generation. Other traits that have been shown to be selected for by long term insectary maintenance are those involved in mating such as, for example, increased size of testes, decrease of accessory glands, and accelerated rotation of the male genitalia [37, 40–42].

With respect to hatching spread, it is noteworthy that no eggs or any strains hatched within the first 24 h post oviposition. The older strains tended to follow what has long been considered the norm for anopheline eggs hatch within 2–3 days post oviposition [20], most likely because older studies focused on colonized strains. However, the more recently colonized VK and Rufisque hatched for up to day 6, suggesting that a longer egg hatching spread may characterize many natural anopheline populations.

The bidirectional selection for early and late egg hatching experiments confirmed the strong heritable component associated with the timing of egg hatching. Using the most recently colonized VK strain and water agitation in all cases, a statistically significant response to selection for early and late egg hatching could be detected as early as the second generation of selection in both replicates.

Despite achieving strong divergent responses in the median time of hatching of early and late hatching strains in all selected strains, egg hatching continued to take place from day 2 to 6 and a span of 5 days. What differed drastically at the end of the selection experiment was the difference in the proportion of eggs that hatched on day 2 or day 5 in early and late strains. These results are in line with those reported by Kaiser et al. [27] who selected early and late hatching lines for six generations which resulted in an 30% increase in the proportion of late hatching larvae [27]. The result of early and late hatching strain crossing experiments suggested that egg-hatching time did not follow a simple Mendelian mode of inheritance [27]. Furthermore, the rate of metabolic activity and embryonic development did not differ between early and late hatching lines [18].

The possibility of selecting for late hatching strains with diapausing eggs could open the door to revised rearing protocols for anopheline strains destined to be used in genetic control programs, provided this does not have unwanted negative effects on their efficacy or safety. Currently, that the eggs of most *Anopheles gambiae* s.l. laboratory strain hatch within 2 days, simplifies some aspects of rearing through improving larval development synchrony. However, a major consequence of fast egg hatching is that is negatively constrains other downstream processes such as the storing and shipping of eggs. For example, the shipping large number of eggs from centralized production centers to local egg-to-adult rearing units would currently be heavily constrained by egg hatching time and survivability. Different egg storage conditions have been explored for storing anopheline eggs for several days before use [33–35], but these methods are not always simple to implement as part of long-distance shipping where the level of humidity, temperature and agitation are difficult to control. The availability of strains with their eggs hatching in 4–5 days without complex handling and storage needs could already greatly facilitate processes of rearing and shipping.

It is important to note that, at this stage, it is not known whether bidirectional selection only caused a shift in programmed duration of diapause or also a change in sensitivity to the spray stimulus in early and late hatching strains. The amount of energy reserve present in eggs is also thought to potentially affect the time at which embryos hatch [16]. Thus, late hatchers may have been selected not only to produce eggs with longer diapause or embryos that require higher rainfall stimulus, but also eggs with more energy reserves to allow the embryo to survive longer in the eggs until the time is right to hatch. Therefore, future studies should explore whether eggs of early and late hatching phenotypes differ in size or mass due to differences in the energy reserves available to the embryos.

Given that our late hatching strains continued to hatch over a range of several days and continued to respond to selection after over 20 generations, it would seem possible to further select for delayed hatching time by adjustment and strengthening of our selection regime. Future studies should also aim at evaluating the fitness of the selected early and late hatching strains. Here, albeit we did not find large differences in the rate of hatch of selected lines, the design of the selection experiment did not allow for fully investigating their fecundity, developmental rate and adult survival and compare those to that of longer-established strains.

## Conclusions

Here we highlighted two important egg hatching traits that characterize natural anopheline mosquito populations but are selected against by long term insectary maintenance. These are the embryonic sensitivity to water agitation and staggered egg hatching. The two combined may enable eggs to hatch with a level of spread that best exploits the great natural variation in larval habitats available to gravid females.

We further demonstrated that eggs hatching time responds to bidirectional selection. The availability of selected late hatching anopheline strains could offer new perspectives to programs aiming to curb malaria via the mass rearing and release of sterile or genetically modified strains of the malaria mosquito *An. gambiae* s.l..

## Data Availability

The data supporting the findings of the study must be available within the article and/or its supplementary materials, or deposited in a publicly available database. Please revise the data availability statement accordingly.

## Abbreviations

ITNs: Insecticide-treated nets
IRS: Indoor residual spraying
SIT: Sterile insect technique
RH: Relative humidity
